# ﻿Nomenclatural notes of *Sabinaconvallium* var. *microsperma* (Cupressaceae)

**DOI:** 10.3897/phytokeys.202.87464

**Published:** 2022-07-21

**Authors:** Yong Yang, Keith Rushforth

**Affiliations:** 1 Co-Innovation Center for Sustainable Forestry in Southern China, Key Laboratory of State Forestry and Grassland Administration on Subtropical Forest Biodiversity Conservation, College of Biology and Environment, Nanjing Forestry University, 159 Longpan Road, Nanjing 210037, China Nanjing Forestry University Nanjing China; 2 Institute of Chartered Foresters, 59 George Street, Edinburgh & The Shippen, Ashill, Cullompton, Devon, EN15 3NL, UK Institute of Chartered Foresters Edinburgh United Kingdom

**Keywords:** Conifer, Cupressacae, nomenclature, Sabinaconvallium var. microsperma

## Abstract

The name Sabinaconvalliumvar.microsperma W.C.Cheng & W.T.Wang was not validly published when it was first described in 1975, but was validated in 1978 at the same time as the specific combination *Sabinaconvallium* (Rehder & E.H.Wilson) W.C.Cheng & W.T.Wang was validly published in *Flora Reipublicae Popularis Sinicae*. Under Art. 41.6 of the Shenzhen Code, other names based onSabinaconvalliumvar.microsperma were valid, including *Sabinamicrosperma* (W.C.Cheng & L.K.Fu) W.C.Cheng & L.K.Fu, Juniperusconvalliumvar.microsperma (W.C.Cheng & L.K.Fu) Silba, *Juniperusmicrosperma* (W.C.Cheng & L.K.Fu) R.P.Adams, despite the reference of the basionym being erroneously cited when these authors made the new combinations.

## ﻿Introduction

When working on an updated catalogue of gymnosperms, we noticed that the small-seeded juniper has nomenclatural problems. The small-seeded juniper was treated either as a species, e.g., *Juniperusmicrosperma* (W.C.Cheng & L.K.Fu) R.P.Adams (Adams 2008, 2014; Shang et al. 2015), *Sabinamicrosperma* (W.C.Cheng & L.K.Fu) W.C.Cheng & L.K.Fu (Fu 1983, in Fl. Xizang. 1: 390), or a variety, e.g.,Sabinaconvalliumvar.microsperma W.C.Cheng & L.K.Fu (Cheng et al. 1975; Wang et al. 1978), Juniperusconvalliumvar.microsperma (W.C.Cheng & L.K.Fu) Silba (Silba 1984; Fu et al. 1999; Farjon 2010). However, it remained ambiguous whether these scientific names used in previous taxonomic works were validly published.

Sabinaconvalliumvar.microsperma W.C.Cheng & L.K.Fu was first recognized and described in Cheng et al. (1975, Acta Phytotax. Sin. 13: 86), but was not validly published in that work under Art. 35.1 of the Shenzhen Code (Turland et al. 2018), because the specific name Sabinaconvallium (Rehder & E.H.Wilson) W.C.Cheng & W.T.Wang was not validly published at that time. W.C.Cheng & W.T.Wang made the new combination *Sabinaconvallium* (Rehder & E.H.Wilson) W.C.Cheng & W.T.Wang (Cheng 1961, in Trees of China, 1: 257), but they did not validly publish the combination under Art. 41.5 because this combination was made after Jan. 1^st^ of 1953 and the authors cited only the basionym but without the reference citation. W.C.Cheng & W.T.Wang unintentionally but validly published the specific combination in Flora Reipublicae Popularis Sinicae (Wang et al. 1978, 7: 372) where they correctly cited the basionym and its reference. In the same work, the varietal name Sabinaconvalliumvar.microsperma W.C.Cheng & L.K.Fu was validated by citing the reference of the protologue (Wang et al. 1978, 7: 373).

There are a few later names based on the invalid name Sabinaconvalliumvar.microsperma W.C.Cheng & L.K.Fu (Cheng et al. 1975). In 1983, W.C.Cheng & L.K.Fu intended to make a new combination *Sabinamicrosperma* (W.C.Cheng & L.K.Fu) W.C.Cheng & L.K.Fu (Fu 1983, Fl. Xizang. 1: 390), but they cited the invalid name Sabinaconvalliumvar.microsperma W.C.Cheng & L.K.Fu as the basionym and its publication in 1975. Silba (1984) made a new combination Juniperusconvalliumvar.microsperma (W.C.Cheng & L.K.Fu) Silba based on the invalid name Sabinaconvalliumvar.microsperma W.C.Cheng & L.K.Fu (Cheng et al. 1975); Adams (2000) treated it as a species in *Juniperus* making the combination *Juniperusmicrosperma* (W.C.Cheng & L.K.Fu) R.P.Adams based on the same invalid basionym. Under Art. 41.6, all these combinations are valid though the reference of the basionym should be corrected.

Adams (2008, 2014) divided Juniperus into three sections, viz. sect. Caryocedrus Endl., sect. Juniperus, and sect. Sabina (Mill.) Spach, and indicated that the three sections can be distinguished using morphological characters, e.g. leaves decurrent or not (decurrent in sect. Sabina vs. jointed in sect. Caryocedrus and *Juniperus*), leaf shape (acicular in sect. Caryocedrus and Juniperus vs. scale-like in sect. Sabina), seed cones size (8–25 mm in sect. Caryocedrus vs. 6–18 mm in sect. Juniperus and *Sabina*), seeds fusion (fused in sect. Caryocedrus vs. free in sect. Juniperus and *Sabina*). Mao et al. (2010) suggested that all of the three sections are monophyletic. Yang et al. (2022) treated *Sabina* as a separate genus from *Juniperus* considering phylogeny, morphology, and utilization purposes. Based on the phylogeny of nuclear markers, Shang et al. (2015) suggested that *Juniperusmicrosperma* is not closely related to *J.convallium*, but sister to a small clade including *J.semiglobosa* and *J.sabina*. Therefore, we treated the generic name *Sabina* and the specific name *Sabinamicrosperma* as accepted.

## ﻿Nomenclature

### ﻿ Sabina

Taxon classificationPlantaePinalesCupressaceae 

convallium (Rehder & E.H.Wilson) W.C.Cheng & W.T.Wang in W.C.Cheng & L.K.Fu, Fl. Reipubl. Popularis Sin. 7: 372 (1978).

43DABC76-1E46-5CC1-8B0D-5AE928BBE496

≡ Juniperusconvallium Rehder & E.H.Wilson in Pl. Wilson. (Sargent) 2(1): 62 (1914); Sabinaconvallium (Rehder & E.H.Wilson) W.C.Cheng & W.T.Wang in W.C.Cheng, Trees of China, 1: 257 (1961) et in W.C.Cheng et al., Acta Phytotax. Sin. 13(4): 76 (1975), *nom. inval*.

#### Type.

**Figure 1.**
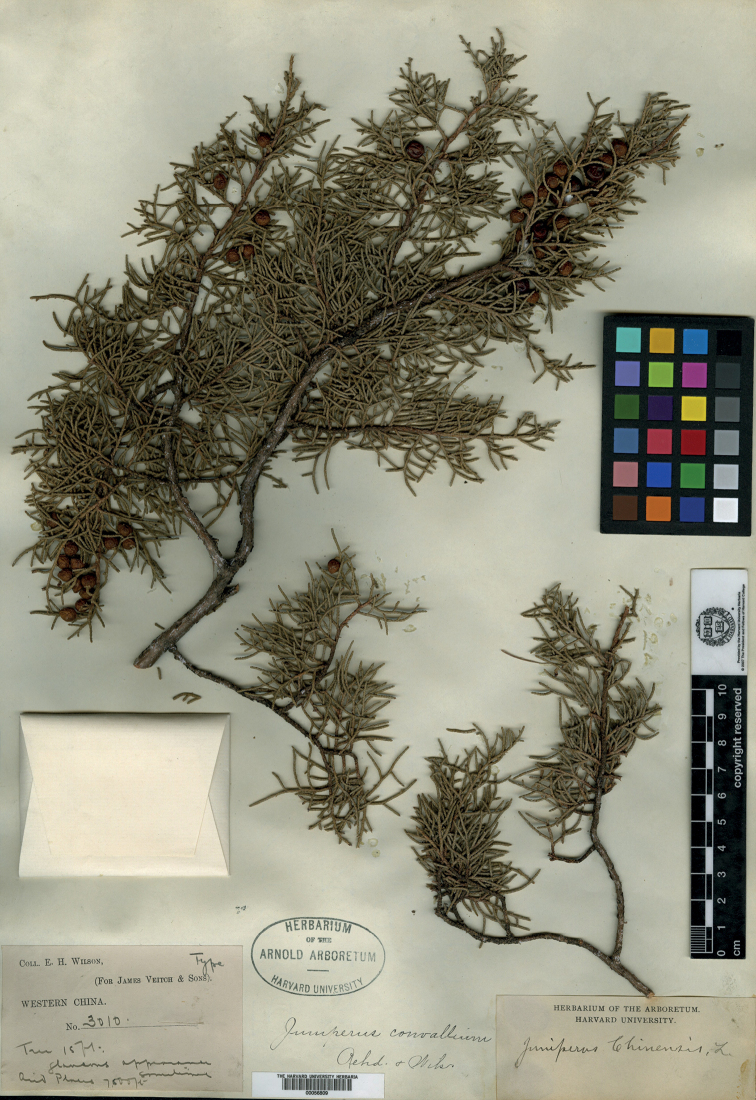
Holotype of *Juniperusconvallium* Rehder & E.H.Wilson: Vietch Exped. 3010 (A00056809).

China (中国). Sichuan (四川), arid places, alt. 2500 m, Aug. 1904, Vietch Exped. 3010 (holotype: A00056809, Fig. 1; isotypes: BM000959921, K001090508, K001090509, K000089628, P00748989, S-C-6511, SYS00001562).

#### Note.

Rehder and Wilson (1914) cited one single collection (Veitch Exped. 3010) in the protologue which should be considered as the type specimen. Adams (2008, 2014) and Farjon (2010) indicated that the specimen in A is the holotype and the isotypes are in BM and K. We found five additional isotypes in international herbaria.

### ﻿ Sabina

Taxon classificationPlantaePinalesCupressaceae 

microsperma (W.C.Cheng & L.K.Fu) W.C.Cheng & L.K.Fu in Fl. Xizang. 1: 390 (1983).

2E01305C-CF87-5899-A49C-AEB392AAF438

≡ Sabinaconvalliumvar.microsperma W.C.Cheng & L.K.Fu in W.C.Cheng et al., Acta Phytotax. Sin. 13(4): 86 (1975), *nom. inval.*; Sabinaconvalliumvar.microsperma W.C.Cheng & L.K.Fu in W.C.Cheng & L.K.Fu, Fl. Reipubl. Popularis Sin. 7: 373 (1978); Juniperusconvalliumvar.microsperma (W.C.Cheng & L.K.Fu) Silba in Phytologia Mem. 7: 33 (1984); Juniperusmicrosperma (W.C.Cheng & L.K.Fu) R.P.Adams in Biochem. Syst. Ecol. 28(6): 540 (2000).

#### Type.

**Figure 2.**
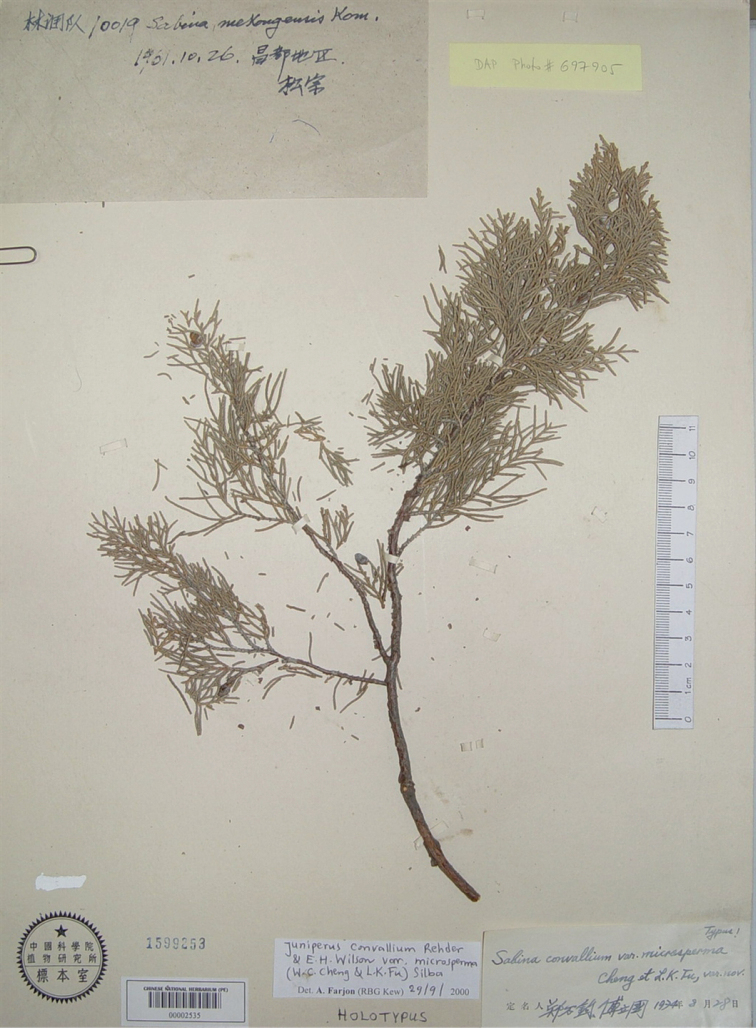
Holotype of Sabinaconvalliumvar.microsperma W.C.Cheng & L.K.Fu: Forestry Exped. 10019 (PE00002535).

China (中国). Xizang (西藏), Qamdu (昌都), Sumzom (松宗), 26 Oct. 1961, Forestry Exped. (森林调查队) 10019 (holotype: PE00002535, Fig. 2).

## Supplementary Material

XML Treatment for Sabina

XML Treatment for Sabina
